# Combining flat‐panel imaging with internal BB markers for precise HDR brachytherapy source localization: A simulation study

**DOI:** 10.1002/mp.70328

**Published:** 2026-02-10

**Authors:** Yibin Ling, Catherine H. Frank, Naoki Dominguez Kondo, Jose Ramos Mendez, Ke Sheng, Qihui Lyu

**Affiliations:** ^1^ Department of Nuclear Engineering University of California Berkeley, CA USA; ^2^ Department of Radiation Oncology University of California San Francisco, CA USA

**Keywords:** brachytherapy, HDR techniques, image‐guided therapy

## Abstract

**Background:**

High Dose Rate (HDR) brachytherapy delivers concentrated radiation to the tumor by placing a high‐dose‐rate radioactive source directly inside the target. However, the sharp dose falloff also increases susceptibility to errors and uncertainties.

**Purpose:**

To enhance source localization accuracy relative to room coordinates and the implant, we optimize the design of a flat‐panel‐based source localization system for HDR brachytherapy.

**Methods:**

Gamma rays from the Ir‐192 Flexisource were simulated with Monte Carlo for image reconstruction. A 50 × 50 cm^2^ flat‐panel detector was placed 15 cm beneath the couch, in which four tungsten ball bearings (BB) are attached. The source location relative to the couch was determined via triangulation. We assessed source localization accuracy based on couch‐BB positions, sizes, BB‐to‐source distance (BSD), and BB‐to‐detector distance (BDD). Since the implant can shift independently, determining the source location relative to the implant, rather than room coordinates, is critical for target dosimetry. To achieve this, a dummy wire containing six tungsten BBs, aligned linearly with increasing separations, was inserted through one of the posterior implanted catheters. The source position relative to the implant was then determined using a rigid transformation that best matched the wire‐BB projections.

**Results:**

Relative to the room coordinates using the couch‐BBs, the proposed system achieves 0.78 mm accuracy. Localization error increases with larger BSD and smaller BDD, ranging from 0.3–2.5 mm (BSD: 10–30 cm) and 0.3–1.2 mm (BDD: 30–10 cm). Introducing implant motion (15 mm translation, 10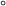 pitch) resulted in 15.8 mm source localization error in room coordinates, while the proposed wire‐BB registration method reduces the error to 1.26 mm relative to the implant.

**Conclusion:**

The study demonstrates the feasibility of using a flat‐panel detector, couch‐attached markers, and a dummy wire for robust localization of the HDR brachytherapy source relative to both the room coordinates and the implant.

## 1 INTRODUCTION

High‐Dose‐Rate (HDR) brachytherapy^1^ is a mainstay treatment option for many types of cancers, including prostate,^2^, ^3^ gynecologic,^4^ breast,^5^, ^6^ skin,^7^ Head and Neck (H&N),^8^ rectal,^9^ and lung^10^, ^11^ cancers. By placing an HDR radioactive source directly inside the treatment target, HDR brachytherapy enables a favorable steep dose gradient when compared to external beam radiotherapy due to the close proximity that amplifies the inverse square effect and attenuation. For the same reason, HDR brachytherapy is sensitive to source locations, as a dose variation of more than 10% per mm can be expected close to the source.^12^ Treatment errors and uncertainties can arise from incorrect transfer tube length measurements, misconnected transfer tubes, catheter reconstruction errors, sliding of transfer tubes, and changes in patient anatomy.^13^, ^14^, ^15^, ^16^ Such inaccuracies can lead to radiation exposure to organs at risk, compromised target coverage, and increased normal tissue toxicity^17^, ^18^, ^19^.

Additionally, because the radioactive source in HDR brachytherapy is placed within or immediately adjacent to the treatment target, it moves synchronously with the target. This intrinsic motion synchronization reduces the impact of internal organ motion compared to external beam radiation therapy, making the treatment more robust against physiological target motion. However, displacement of implanted catheters or applicators can affect dose distribution to organs‐at‐risk (OARs). For example, a catheter displacement of a few millimeters near sensitive structures such as the rectum or bladder can substantially alter the delivered dose, resulting in increased toxicity risks.^20^, ^21^ Thus, meticulous monitoring and accurate identification of internal target motion are essential to ensure treatment precision and safety.

Despite the paramount necessity of treatment monitoring for brachytherapy, independent verification systems for tracking treatment dosimetry are currently unavailable in the clinic. Various methods have been proposed to monitor HDR brachytherapy treatment, including in vivo dosimetry (IVD)^22^, ^23^, ^24^ and electromagnetic tracking (EMT) technology.^25^, ^26^ However, these methods do not completely address the source localization error. For example, the EMT technology tracks the position and orientation of a miniaturized sensor coil placed at a needle tip^25^ to automatically reconstruct the catheter location before HDR treatment. EMT improves catheter reconstruction accuracy but does not measure the source location during treatment. The IVD method requires manually inserting a dosimeter probe into applicators placed in critical organs such as the rectum and bladder, using radiation dosimeters such as optical fiber,^27^ thermos‐luminescent dosimeters (TLD)^28^, ^29^ or semiconductor diodes.^30^, ^31^ In addition to the potential introduction of added risks, discomfort, and extra workload, the IVD point dose measurement is sensitive to the detector positioning in the high dose‐gradient fields, where a moderate spatial uncertainty can translate to much higher dosimetric uncertainty, making reliable interpretation of the results challenging.

Treatment verification using external image panels has recently been brought up as a direct verification of the source position. In 2017, Fonseca et al.^32^ used an external imaging panel to acquire the exit photons from an HDR ^192^Ir source within a water phantom as a pretreatment verification approach. The source location was determined through an exponential fit of the detector response. In 2023, Wagenberg et al.^33^ used an external imaging panel to experimentally capture the exit photons of ^192^Ir. Using radiopaque markers at fixed locations, they successfully determined the source location with an average accuracy of 1.7 mm, assuming a static phantom. However, the source location relative to the patient's anatomy was determined using external markers attached to the patient's surface. This approach is limited by the low marker placement reproducibility and distinct motion patterns between external markers and internal organs.^34^, ^35^ Consequently, the published method could not account for internal anatomical changes that may shift the implant relative to the patient's external surface. Such a shift alone does not change the target dose, since the catheters are fixed to the implant and move together with the target. This should be differentiated from a dwell position error. The current study aims to overcome these challenges by optimizing the geometry and using a combination of external couch‐BBs to define the source in room coordinates and internal wire‐BBs to capture implant motion relative to the room.

## 2 METHODS

### 2.1 Coordinate system

**FIGURE 1:**
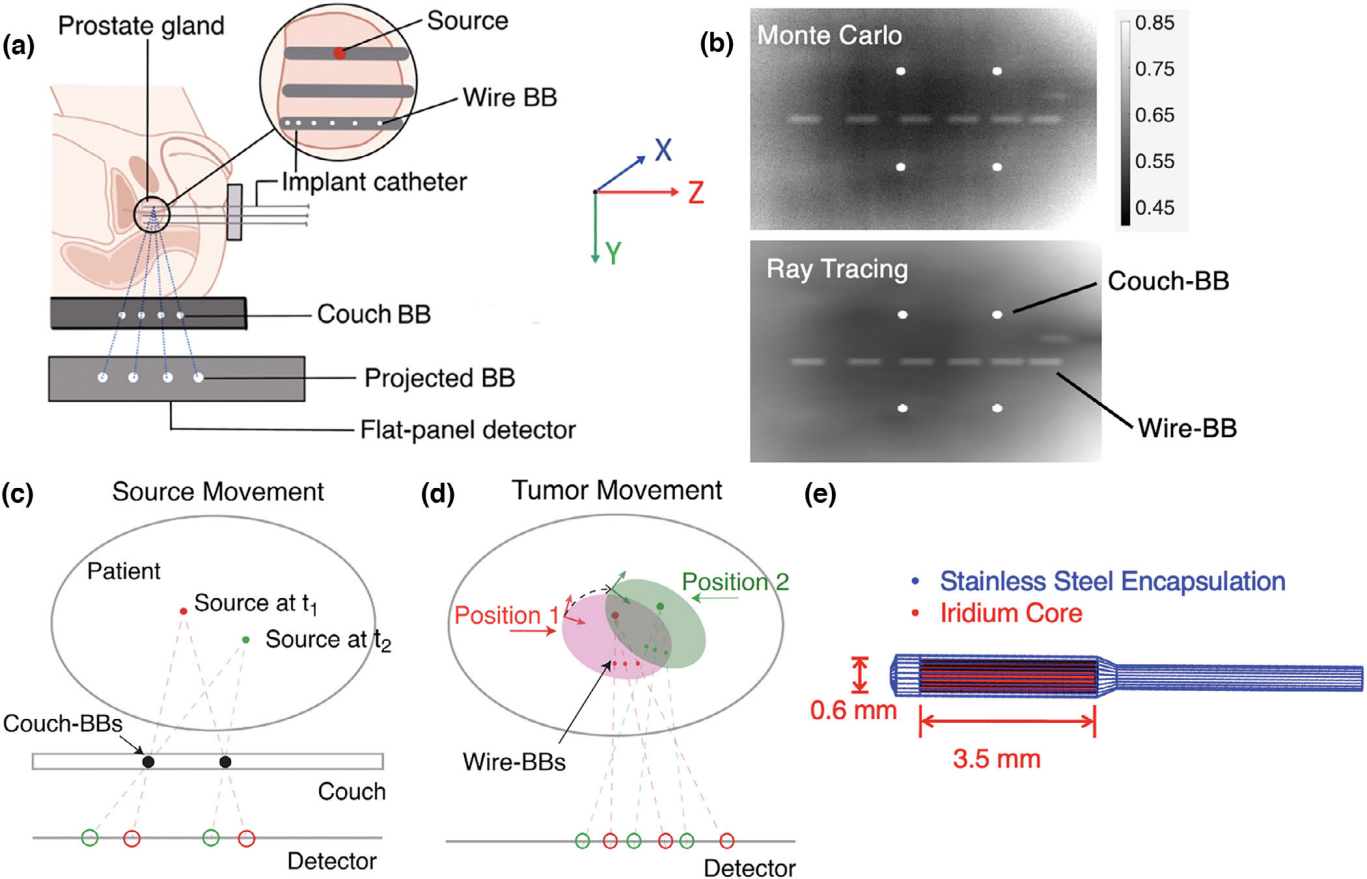
(a) Schematic diagram of the brachytherapy source position localization system. The patient couch with attached tungsten BB markers, ^192^Ir source, inserted wire‐BBs and the flat‐panel detector are illustrated. (b) Contrast‐adjusted projection image of couch‐BBs and wire‐BBs generated by 10^11^ histories simulated in OpenTOPAS (top) and ray tracing (bottom). A prostate CT dataset was used to model patient attenuation. (c) Source localization in room coordinates through triangulation. (d) Rigid transformation of target, source and wire‐BB positions. (e) ^192^Ir source geometry.

Our proposed source monitoring system is depicted in Figure 1a. The system employs two primary coordinate systems: The room coordinate system and the implant coordinate system. The room coordinate system consists of four tungsten ball bearings (BBs) attached to the treatment couch, referred to as couch‐BBs. Tungsten BBs are high‐density, spherical fiducial markers, commonly used in external beam radiotherapy for localization. Since the treatment couch remains relatively fixed within the room, it serves as the reference for the room coordinate system. The room coordinate system was defined with its origin at (0,0,0). The *X*‐ and *Z*‐axes were parallel to the detector plane, while the *Y*‐axis was normal to the detector plane. The implant coordinate system, on the other hand, defines the source position relative to the implant. The dummy wire, consisting of six tungsten BBs, is inserted into the patient through one of the implanted catheters and referred to as wire‐BBs. The wire‐BBs are secured to the catheter and assumed to move together with the implant. A flat‐panel detector placed beneath the couch collects the exit gamma ray photons emitted by the HDR ^192^Ir source. Due to their high‐density, both couch‐BBs and wire‐BBs attenuate the ^192^Ir gamma rays substantially, creating visible shadows on the projection images, as shown on the Monte Carlo simulation (10^11^ histories) and ray‐tracing calculation, respectively (Figure 1b). Accurate localization of the source requires determining its position within both the room and the implant coordinate systems and quantifying the relative shift between them.

### 2.2 BB localization on the projection

Shadows of the couch‐BB and wire‐BB are identified on the projection images through the following image processing steps. First, large‐scale intensity variations are removed using top hat filtering with a disk‐shaped structuring element with a radius of 10 pixels. This background removal process enhances small, localized bright objects, for example, BBs. The filtered image is then converted into a binary representation that retains only features matching the structuring element. Morphological erosion is applied to remove thin lines and small isolated objects smaller than 3 pixels. Connected components in the binary image are identified to isolate individual BBs, and their outer boundaries are traced to calculate their respective areas. Circularity is then computed as:

$$C=\frac{4\pi \cdot \mathrm{Area}}{\mathrm{Perimete}r^{2}}.$$

When circularity equals 1.0, it indicates perfect circles. Couch‐BBs are identified as the regions with a high circularity value (greater than 0.9), and wire‐BB shadows are identified with a different circularity threshold (0.3 to 0.8) because wire‐BBs are smaller and closer to the source, creating elongated shadows on the projections. BB centroids were computed as their center of mass.

### 2.3 Accuracy of localizing the source in room coordinates using couch‐BBs

The source localization within the room coordinate system utilizes the couch‐BBs. By analyzing the locations of the couch‐BB shadows and their known physical positions, the source location can be determined through triangulation, as illustrated in Figure 1c. We connect lines between the projected couch‐BB centers and their actual physical locations. Ideally, these lines intersect at the source location for a point source model. However, these lines do not intersect perfectly due to limited detector resolution, imaging noise, imperfect BB segmentations, and line source geometry. We locate the source as the point that minimizes the total distance to all lines. Mathematically, this problem can be expressed as minimizing:

$$\min_{p}\;\sum_{i}\;d\left(p,l_{i}\right)$$

where p is the source position, $l_{i}$ is the *i*‐th line defined by the BB projections, and $d(p,l_{i})$ is the shortest distance from p to the line $l_{i}$. This approach enables real time monitoring of the source position in the room coordinate system.

### 2.4 Accuracy of localizing the relative shift between the implant and room coordinates using Wire‐BBs

To determine the shift between the room coordinate system relative to the implant coordinate system due to implant motion, we introduce the wire‐BBs, which consist of six 0.5 mm radius BBs, separated by increasing distances of 4, 4.5, 5, 5.5, and 6 mm. This unique spacing pattern encodes the BB sequencing on the projection image. Spacings were selected to prevent overlap in BB shadows while minimizing the total wire length. Because the wire‐BB spacing is greater than the source length, overlap is prevented regardless of the relative positions of the wire‐BBs and the source. In the envisioned clinical workflow, the dummy wire containing the six tungsten BBs would be inserted into one of the implanted catheters prior to the planning CT acquisition. The wire‐BB should remain secured to the implanted catheter from the planning CT through the initial phase of treatment delivery, during which all catheters except the one containing the wire‐BB are delivered. In this phase, the wire‐BB‐bearing catheter is assumed to be rigid and move along with the treatment target (Figure 1d). We will determine the room‐to‐implant registration based on the wire‐BB projections in this phase. Once this transformation is established, the wire‐BBs can be safely removed before continuing treatment with the remaining dwell positions. In this simulation study, the 3D geometry of the wire‐BBs were modeled with a slight curvature (radius of 500 mm curvature) to mimic the trajectory of an implanted catheter. The corresponding patient CT voxels were overwritten with tungsten material in the Monte Carlo simulation.

From the wire‐BB projections, we can determine the 3D positions of the wire‐BBs in both the room and the implant coordinates by solving an optimization problem. Each wire‐BB lies on a line connecting the source position s to its shadow $p_{\mathrm{shadow},\;i}$ on the detector. The parametric equation of the line is given by:

$$p_{i}=s+d_{i}\frac{p_{\text{shadow},\;i}-s}{\left|p_{\text{shadow},i}-s\right|},$$

where $p_{i}$ is the location of the *i*‐th wire‐BB, and $d_{i}$ is the unknown distance from the source to the i‐th BB. Since BBs follow the rigid catheter, their positions can also be described by a rigid transformation:

$$\;p_{i}=R\cdot p_{\mathrm{CT},\;i}+t$$

where R is the rotation matrix, t is the translation vector and $p_{\mathrm{CT},\;i}$ are the known wire‐BB positions from the planning CT image.

The optimization problem for wire‐BB registration is then formulated as:

$$\min_{R,\;t,\;d_{i}}\sum_{i}{\left\|\left(R\cdot p_{\mathrm{CT},\;i}+t\right)-\left(s+d_{i}\frac{p_{\mathrm{shadow},\;i}-s}{\left|p_{\mathrm{shadow},\;i}-s\right|}\right)\right\|}^{2}$$

$$\mathrm{subject}\;\mathrm{to}\;\;d_{i}\geq 0\;,$$

This minimizes the error between the rigidly transformed BB and parametric positions. Using an iterative optimization algorithm (Broyden–Fletcher–Goldfarb–Shanno), we determine the optimal R,t, and $d_{i}$ values for all wire‐BBs. The obtained transformation precisely encodes the relative shift between implant and room coordinates.

### 2.5 Accuracy of localizing the source in implant coordinates using both couch and Wire‐BBs

Once the rigid transformation is determined from the above optimization, we apply the same rotation and translation to the source position calculated from the room coordinate system to transform it into the implant coordinate space. This process ensures that the HDR source localization is accurately registered within the anatomical frame of reference and differentiates the implant shift from source positioning errors.

### 2.6 Projection simulation

The projection data is created using Monte Carlo simulation with the OpenTOPAS toolbox.^36^, ^37^ The Ir‐192 Flexisource model was used, with full modeling of the source capsule and core (3.5 mm length and 0.6 mm diameter) (Figure 1e). Photon emission was simulated isotropically in all directions using the OpenTOPAS Volumetric brachytherapy source type,^37^, ^38^ which randomly samples emission points within the active source volume and emits photons uniformly over 4*π* steradians. The photon energy spectrum was sampled from the discrete, non‐monoenergetic emissions of Ir‐192.^38^, ^39^ A 1 cm thick carbon fiber treatment couch with a density of 0.8 g/cm^3^ was included. For each configuration, we simulated 10^10^–10^11^ primary particles depending on the Signal‐to‐Noise Ratios (SNR), which would be emitted by a clinical HDR source within sub‐seconds. The flat‐panel detector was modeled as a 50 cm × 50 cm scoring plane with 500 × 500 pixels with a resolution of 1.0 mm. In this study, we did not simulate detector response for simplicity. We used Monte Carlo simulation to directly record the photon fluence in air at the detector plane. A clinical prostate CT dataset (512 × 512 × 132 voxels, voxel size 0.98 × 0.98 × 3 mm^3^) was used for attenuation modeling in all simulations.

#### 2.6.1 Simulation1: Effect of couch‐BB pattern on source localization accuracy in room coordinates

In this section, we isolated the impact of the couch‐BB geometric layout while keeping imaging geometry fixed. The couch‐BB radius was 5 mm, with BDD and BSD both 15 cm (a constant source‐to‐detector distance SDD at 30 cm). We evaluated five layouts with *N* = 2, 3, 4, 5, 6 BBs and five fixed‐BB layouts with spacing from 12 mm to 60 mm.

#### 2.6.2 Simulation 2: Effect of couch‐BBs size, BSD and BDD on source localization accuracy in room coordinates

We varied BSD from 10 cm to 30 cm and the couch‐BB radii from 2 mm to 5 mm, while fixing the detector at 40 cm from the source. Additional simulations varied the BDD from 10 cm to 30 cm while keeping the couch‐BB radius at 5 mm and testing BSD values of 10, 15, and 20 cm. These configurations isolated the effects of BB size, BSD, and BDD on source localization accuracy and field‐of‐view (FOV). We computed FOV using:

$$\mathrm{FOV}\;=2\left[\left(D-\left(a+r\right)\right)\frac{\mathrm{BSD}}{\mathrm{BDD}}-\left(a+r\right)\right]$$

where D is detector half‐width (25 cm), a is BB pattern half‐width (for a 6 × 6 cm^2^ square, a = 3cm), and *r* is BB radius (5 mm).

#### 2.6.3 Simulation3: Source shift in different directions

We evaluated the robustness of source localization under different source positions. The source was initially positioned at the center of the room coordinate system (0, 0, 0), 15 cm above the couch‐BBs (radius = 5 mm), with a detector 15 cm below (SDD = 30 cm). The source was then shifted along the *X* and *Z* axes within *a* ± 5 cm range to test the impact of parallel position shifts on localization accuracy.

#### 2.6.4 Simulation4: Implant motion

To systematically assess the impact of internal target motion on source localization accuracy, we simulated a series of clinically relevant rotations and translations based on reported prostate intra‐fraction motion. Specifically, we introduced rotations of 1°, 2°, 5°, and 10° around the left‐right (LR) axis, combined with translations ranging from 1 mm to 15 mm along superior‐inferior (SI) direction. These motion ranges are consistent with previous studies, which report prostate rotations of ∼5° (occasionally up to 10°), and translations typically < 5 mm, rarely reaching 15 mm.^40^, ^41^, ^42^ To reduce computation time, the simulated motion was implemented using a ray tracing forward projection method,^43^ which had been validated against Monte Carlo simulations.

### 2.7 Performance evaluation metrics

The performance of the proposed detection system was evaluated using two metrics: source localization accuracy and projected couch‐BB deviation. Source localization accuracy measures the Euclidean distance between the calculated and actual source positions, reflecting the system's precision in determining the source location. Projected couch‐BB deviation quantifies the Euclidean distance between the detected couch‐BB center positions on the detector and their ground truth projections. The ground truth is determined by drawing a straight line from the known simulated source center to the known couch‐BB center and identifying where this line intersects the detector plane. This metric assesses the system's capability to accurately reconstruct BB positions on the detector, which is critical for precise source localization.

## 3 RESULTS

### 3.1 Effect of couch‐BBs pattern on source localization accuracy in room coordinates

**FIGURE 2:**
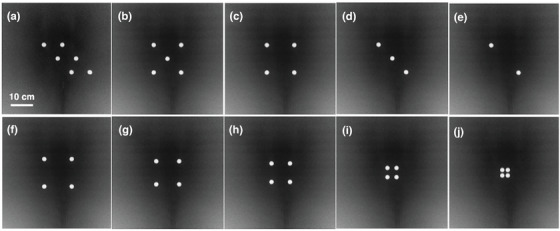
Projected images of different couch‐BB numbers and patterns from OpenTOPAS simulation.

Figure 2 shows the projection images generated using Monte Carlo simulation with 10^10^ histories for different couch‐BB numbers and patterns. The mean relative statistical uncertainty of the projection image was 2.3%. Computation time was approximately 20 h on 128 CPU threads per projection. As shown in Table 1, we tested the effect of couch‐BB geometric distribution and the number of couch‐BBs on the source localization error. The source localization error ranges between 0.48 mm and 0.54 mm across all configurations, indicating that the number of couch‐BBs does not significantly impact localization accuracy.

**TABLE 1:** Effect of couch‐BB numbers on source localization accuracy. Projected couch‐BB deviation measures the difference between the projected couch‐BB center locations on the detector and their ground truth projections.

Number of BBs	Source localization accuracy (mm)	Projected couch‐BB deviation (mm)	Projection image
6	0.48	0.18	Figure 2a
5	0.50	0.16	Figure 2b
4	0.50	0.20	Figure 2c
3	0.54	0.19	Figure 2d
2	0.54	0.20	Figure 2e

Table 2 shows the source localization accuracy and deviation as the couch‐BB spacing was reduced from 60 mm to 12 mm. The source localization error remains stable (0.5 mm–0.6 mm) as the couch‐BB spacing decreases from 60 mm to 20 mm, but shows a sharp decline in accuracy when reduced further to 12mm spacing. This indicates that reducing the spacing below 20 mm significantly compromises localization accuracy, likely due to insufficient geometric spread limiting triangulation accuracy.

**TABLE 2:** Effect of couch‐BB spacing on source localization accuracy. Projected couch‐BB deviation measures the difference between the projected couch‐BB center locations on the detector and their ground truth projections.

Distance between BBs (mm)	Source localization accuracy (mm)	Projected couch‐BB deviation (mm)	Projection image
60	0.59	0.20	Figure 2f
50	0.51	0.18	Figure 2g
40	0.54	0.18	Figure 2h
20	0.59	0.09	Figure 2i
12	1.78	0.14	Figure 2j

### 3.2 Effect of couch‐BBs size, BSD and BDD on source localization accuracy in room coordinates

**FIGURE 3:**
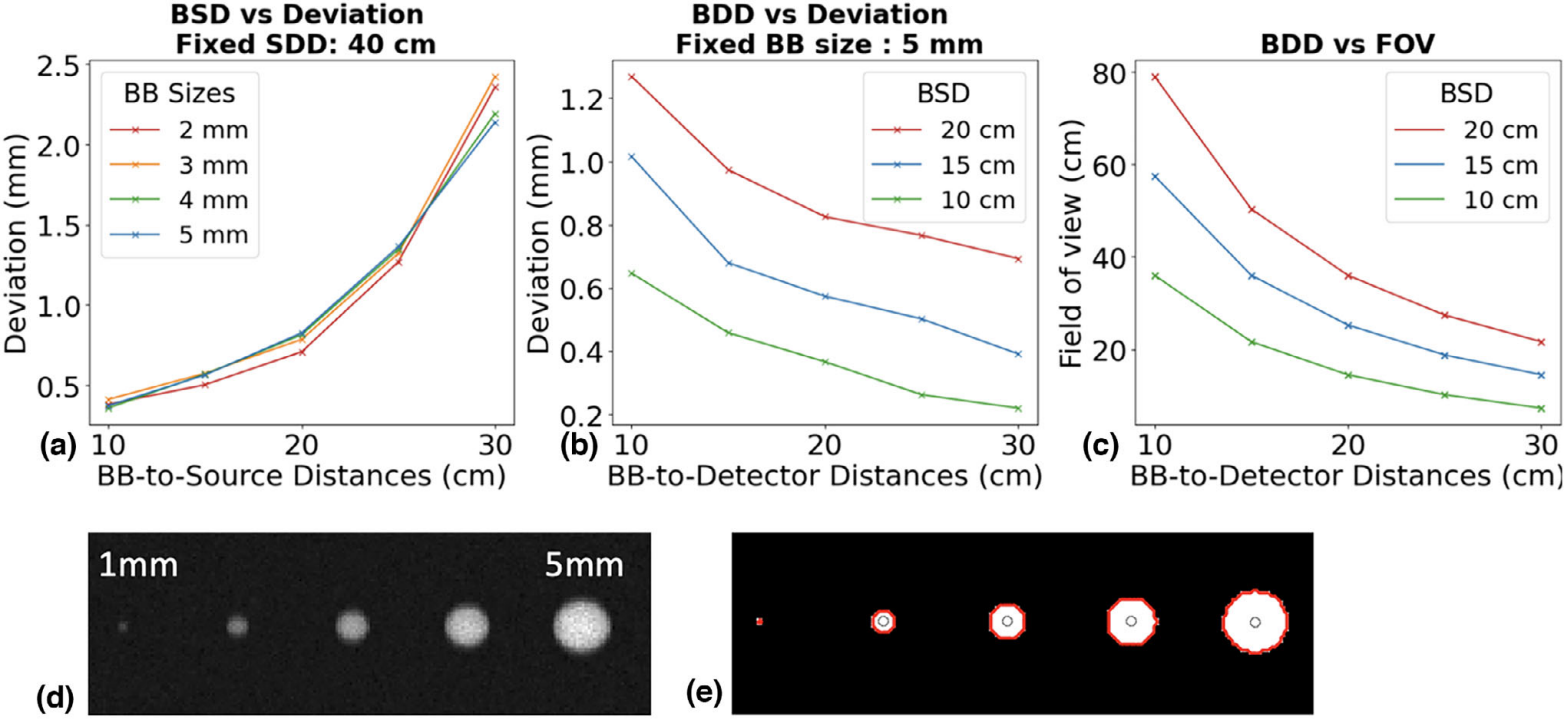
(a) Effect of BB‐to‐source distance (BSD) on the source localization error for different BB sizes. (b) Effect of BDD on the source localization error for different BSDs. (c) Effect of BDD on the FoV for different BSDs. (d) Monte Carlo simulation (10^10^ histroies) of BB projections with sizes ranging from 1–5 mm radius (e) Segmented projection image with detected BB boundaries and centroids corresponding to (d).

Figure 3a shows the effect of BSD and couch‐BB sizes on the source localization error. The deviation increases from 0.36–0.41 mm to 2.14–2.62 mm as BSD increases from 10 cm to 30 cm. Changing couch‐BB radii from 2 mm to 5 mm only results in an average difference of 0.2 mm in source localization error at fixed BSDs. As illustrated in Figure 3d, 1 mm BBs exhibit a low contrast to noise ratio and therefore cannot be accurately detected.

Figure 3b shows the effect of BDD on the source localization error with couch‐BB sizes fixed at 2 mm. For BSD fixed at 10 cm, 15 cm, and 20 cm, the source localization error reduces from 0.65–1.33 mm to 0.22–0.56 mm as the BDD increases from 10 cm to 30 cm. However, increasing the BB‐to‐detector distance (BDD) reduces the FOV. For example, using the BB pattern in Figure 2c, at BSD = 10 cm, a BDD of 10 cm provides a FOV of 36 × 36 cm, while increasing the BDD to 30 cm reduces the FOV to 7.3 × 7.3 cm. This trade‐off suggests the need to optimize the BDD to balance accuracy and FOV for each treatment plan. Figure 3c visually illustrates the impact of varying BDD and BSD configurations on the resulting FOV, providing valuable guidance for selecting optimal settings for individual cases.

### 3.3 Source shift in different directions in room coordinates

In this simulation, the source is positioned at the center (0, 0, 0), 15 cm away from the 5 mm‐radius couch‐BBs, with a BDD of 15 cm. When the source is within the range of ± 5 cm from the center, the source localization accuracy ranges from 0.5 mm to 0.6 mm, as shown in Table 3. This result demonstrated a robust performance of the proposed localization method as the source moves parallel to the detector.

**TABLE 3:** Verification of couch‐BB pattern coverage and source position accuracy under various source movements parallel to the detector.

Source location (cm)	Source localization error in (*x*, *y*, *z*) (mm)	Source position accuracy (mm)	Projected couch‐BB deviation (mm)
(0, 0 0)	(0.02, 0.50, 0.04)	0.50	0.20
(5, 0, 5)	(0.09, 0.50, 0.19)	0.56	0.34
(5, 0, −5)	(0.12, 0.50, 0.12)	0.54	0.25
(−5, 0, 5)	(0.07, 0.50, 0.13)	0.52	0.37
(−5, 0, −5)	(0.05, 0.51, 0.14)	0.55	0.37

### 3.4 Transferring source position in room coordinates to implant coordinates

In this simulation setup, we placed the dummy wire approximately 3 cm beneath the source, with the detector positioned 30 cm below the source. Couch‐BBs were positioned 15 cm above the detector. Without introduced target motion, the simulation yielded a baseline source localization accuracy of 0.78 mm in room coordinates.

We introduced rotations of 1°, 2°, 5°, and 10° around the LR axis, combined with translations ranging from 1 mm to 15 mm along SI direction (Table 4). Table 4 shows that larger rotations and translations generally resulted in greater deviations in source localization accuracy relative to implant coordinates. For instance, a 10° rotation combined with a 15 mm translation led to a localization deviation of 1.26 mm within the implant. In contrast, smaller motions, such as a 1° rotation with a 1 mm translation, produced a localization deviation of 0.93 mm.

**TABLE 4:** Performance of the source localization method under varying simulated rotations around the LR axis and translational shifts along the SI axis. Each scenario compares recovered rotation angles and translation vectors to the ground truth. Localization accuracies are reported in both implant and room coordinates.

Rotation / Translation	Recovered rotation / Translation	Localization accuracy in room coordinate (mm)	Localization accuracy in implant coordinate (mm)
1° / 1 mm	0.2° / 1.2 mm	1.77	0.93
2° / 2 mm	1.3° / 2.2 mm	2.85	1.00
5° / 5 mm	4.7° / 5.2 mm	6.10	1.17
10° / 10 mm	9.8° / 9.9 mm	11.6	1.08
10° / 15 mm	10.4° / 14.8 mm	15.8	1.26

**FIGURE 4:**
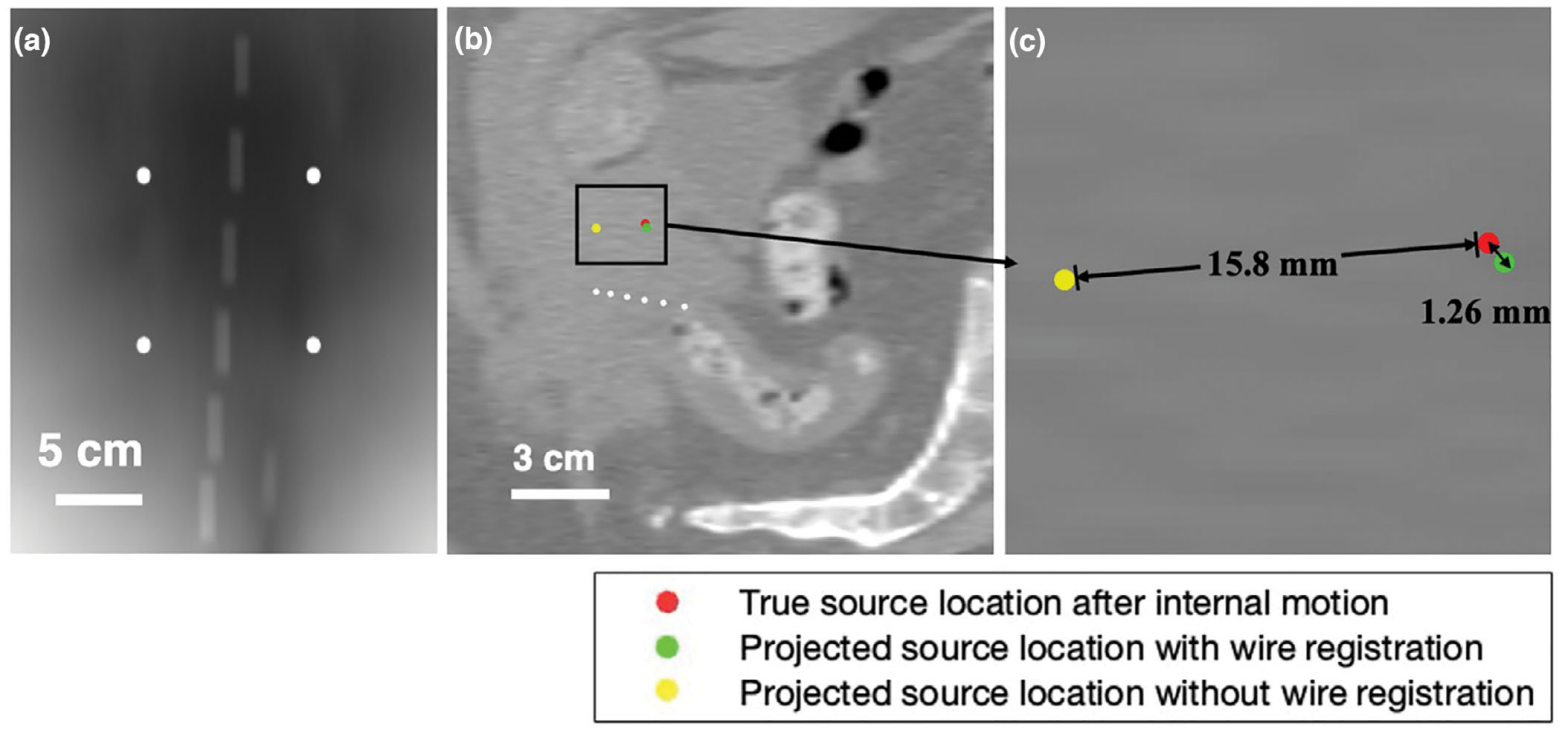
(a) Projection image of tilted wire from ray tracing. (b) Contrast‐adjusted CT slice sagittal image showing wire‐BBs, source location, and projected source locations. (c) Zoomed in view highlighting position deviations.

Figure 4 illustrates the effect of this simulated internal motion on source localization. Figure 4a presents the 2D forward projection of both couch and wire‐BBs under a scenario of 10° rotation and 15 mm translation. Figure 4b,c show sagittal and zoomed in views comparing projected source positions with and without wire‐BB registration. Without wire‐BB registration, the projected source location is 15.8 mm from the true source location. With wire‐BB registration, the source location can be determined within 1.3 mm from the true source location. This demonstrates significantly improved source localization accuracy in the implant coordinates under patient motion.

## 4 DISCUSSION

HDR source location verification using a flat‐panel has been recently demonstrated.^32^, ^33^ The method triangulates source location with the Ir‐192 gamma rays and externally placed BBs. The method shows the promise of real time source localization for treatment monitoring. However, the optimal imaging geometry, placement of the BBs, and BB sizes have not been studied. Moreover, previous studies either localized the source relative to the couch or to the patient surface, missing the critical fact that the HDR dosimetry is determined by the relative source location to the implant. In this study, we systematically analyzed the impact of geometry factors and BB configurations on HDR source localization error, optimized relevant geometric parameters, and developed novel internal placement of markers for dosimetrically relevant source localization. As illustrated in Figure 3, localization accuracy in room coordinates primarily depends on the distances among couch‐BB markers, the source, and the detector. Specifically, accuracy improves as couch‐BB markers are placed closer to the source when the detector remains at a fixed distance from the patient. This improvement arises from an increased magnification factor of the projected BBs. Similarly, localization accuracy benefits from an increased distance between the detector and the couch‐BB markers, but at the expense of a reduced FOV. Notably, better than 0.5 mm localization accuracy was achieved when the BSD was under 15 cm and the BDD exceeded 25 cm, corresponding to a maximum FOV of approximately 20 × 20 cm.

In contrast, couch‐BB size has little effect on source localization accuracy for BBs when the radius exceeds 2 mm. Increasing the radius from 2 mm to 5 mm changes accuracy by less than 0.2 mm. Consistent with prior work, we observed that 1 mm BBs are difficult to detect on projection images. In principle, only two couch BBs are needed for the triangulation method to determine the source position. Adding additional BBs does not significantly improve localization accuracy but may increase the robustness of the algorithm through the averaging effect and outlier rejection. Similarly, as shown in Table 2, larger in‐plane spacing between BBs also improves triangulation robustness and reduces angular uncertainty.

In HDR brachytherapy, the Ir‐192 source typically has an initial activity of ∼10 Ci that decays to be ∼3 Ci before a new source is installed, which produces at least 2×10^11^ photons per second. As shown in Figure 3d, projections simulated with 10^10^ photons already provide a sufficient signal‐to‐noise ratio for reliable couch‐BB detection. This corresponds to less than 0.05 s of emission time for a clinical Ir‐192 HDR source, suggesting real time source localization is feasible. Respiratory motion within such a short period is expected to be minimal, and the motion blurring from respiration should have little effect on BB visibility or localization accuracy.

Under optimal geometric conditions, our approach achieved submillimeter accuracy, outperforming previous external imaging‐based methods. Wagenberg et al.^33^ reported an average localization accuracy of 1.7 mm using external radiopaque markers placed on the patient's skin in experimental measurements. While our study is supported by Monte Carlo simulation results and does not account for setup uncertainties inherent in experiments, the results demonstrate that localization error is strongly influenced by geometry, ranging from 0.5 mm to over 2 mm. Optimizing the setup configurations provides improved differentiation of minor inter‐dwell positional errors, for example, 2 mm. This difference is clinically meaningful in HDR brachytherapy, where dose gradients can exceed 10% per millimeter near the source.^12^ Additionally, the method by Wagenberg et al. relies on external markers, which are unreliable surrogates of internal organ motions. In comparison, our proposed internal wire‐BB registration computes internal target motion, and achieves 1.3 mm source localization accuracy even with significant internal organ motion. It should be noted that our reported localization accuracy represents an estimate obtained under idealized simulation conditions. Additional factors such as detector noise and setup uncertainties were not considered in this study.

We positioned the wire‐BBs approximately 3 cm beneath the source, simulating placement within one of the posterior brachytherapy catheters. While this placement inherently limits direct verification for nearby posterior channels, this limitation is clinically manageable. Brachytherapy procedures typically last around 10 min, during which significant internal organ movement is unlikely. Our recommended clinical workflow involves verifying anterior channels first to establish the transformation between implant and room coordinates. Once this transformation is established, the wire‐BBs can be safely removed, and the verified coordinates can guide subsequent dwell positions.

Accurate and consistent alignment between room and implant coordinate systems is essential, as even minor variations in internal organ positions can significantly affect delivered dose distributions. For small target motions or shifts, localization accuracy between these coordinate systems remains stable. However, larger target motions introduce notable discrepancies. Significant deviations in room coordinates may not necessarily indicate errors in source positioning or catheter movement but may instead reflect substantial target motion. In contrast, large deviations detected within implant coordinates suggest potential treatment errors, requiring immediate intervention. Physically, such errors may arise from incorrect transfer tube length measurements, misconnected or mismatched transfer tubes, catheter reconstruction inaccuracies, or mechanical issues such as catheter displacement or sliding of transfer tubes—all of which can lead to source misplacement, underdosing of the tumor, or unintended exposure of OARs.^13^, ^14^, ^15^, ^16^

We expect that catheters move synchronously with internal targets, relying on the assumption that the tumor behaves as a single rigid body. By securing the wire‐BBs to the implanted catheters, displacement of the wire‐BBs from the implant is unlikely. In rare cases where displacement does occur, it can be detected using two independent methods to compute the rigid transformation from room to implant coordinates: (1) comparing the source trajectory derived from the projection with the planning CT, and (2) using the wire‐BBs as described in Method Section 5. Under normal conditions, both methods should yield consistent rigid transformation matrices. An inconsistency between the two indicates wire‐BB shifts or source positioning errors. Under rare scenarios when these errors happen, the proposed method can detect these errors, but the current rigid transformation method may not quantify the extent of the wire‐BB shifts.

In addition, adjacent tissues or distinct regions within the tumor can deform or move independently due to respiration or other physiological processes. Currently, our technique cannot differentiate such nonrigid motion, potentially resulting in unintended dose variations to OAR and compromising treatment planning accuracy. Future advancements in monitoring of OAR motion, alongside adaptive planning techniques, could effectively overcome these limitations by providing enhanced motion tracking and enabling more precise dose adjustments.

Although we tested our algorithm on a prostate case, the same method is applicable to other anatomical sites treated with interstitial catheters. For brachytherapy using applicators, it is important to account for scattering and attenuation effects, particularly with intracavitary applicators made of titanium alloys, such as Tandem and Ovoid (T&O) or Tandem and Ring (T&R). These scenarios will require additional Monte Carlo simulations tailored to each applicator geometry.

## 5 CONCLUSION

This study demonstrated the feasibility of using a single flat‐panel detector and tungsten BBs for precise localization of brachytherapy sources and using internal wire‐BBs for anatomical mapping.

## CONFLICT OF INTEREST STATEMENT

The authors declare no conflicts of interest.

## Data Availability

The data that support the findings of this study are available from the corresponding author upon reasonable request.
